# [^18^F]MPPF and [^18^F]FDG μPET imaging in rats: impact of transport and restraint stress

**DOI:** 10.1186/s13550-020-00693-3

**Published:** 2020-09-29

**Authors:** Verena Buchecker, Ann-Marie Waldron, R. Maarten van Dijk, Ines Koska, Matthias Brendel, Barbara von Ungern-Sternberg, Simon Lindner, Franz Josef Gildehaus, Sibylle Ziegler, Peter Bartenstein, Heidrun Potschka

**Affiliations:** 1 Institute of Pharmacology, Toxicology and Pharmacy, Ludwig-Maximilians-University, Königinstr. 16, 80539 Munich, Germany; 2 Department of Nuclear Medicine, University Hospital of Munich, Ludwig-Maximilians-University, Munich, Germany

**Keywords:** Distress, 5-HT_1A_ receptor, Brain glucose metabolism, Micro-PET, Behavior, Animal model

## Abstract

**Background:**

Stress exposure can significantly affect serotonergic signaling with a particular impact on 5-HT_1A_ receptor expression. Positron emission tomography (PET) provides opportunities for molecular imaging of alterations in 5-HT_1A_ receptor binding following stress exposure. Considering the possible role of 5-HT_1A_ receptors in stress coping mechanisms, respective imaging approaches are of particular interest.

**Material and methods:**

For twelve consecutive days, Sprague Dawley rats were exposed to daily transport with a 1 h stay in a laboratory or daily transport plus 1 h restraint in a narrow tube. Following, animals were subjected to μPET imaging with 2′-methoxyphenyl-(N-2′-pyridinyl)-p-[^18^F]fluoro-benzamidoethylpiperazine ([^18^F]MPPF) and 2-deoxy-2-[^18^F]fluoro-D-glucose ([^18^F]FDG). Behavioral and biochemical parameters were analyzed to obtain additional information.

**Results:**

In rats with repeated transport, hippocampal [^18^F]MPPF binding exceeded that in the naive group, while no difference in [^18^F]FDG uptake was detected between the groups. A transient decline in body weight was observed in rats with transport or combined transport and restraint. Thereby, body weight development correlated with [^18^F]MPPF binding.

**Conclusions:**

Mild-to-moderate stress associated with daily transport and exposure to a laboratory environment can trigger significant alterations in hippocampal binding of the 5-HT_1A_ receptor ligand [^18^F]MPPF. This finding indicates that utmost care is necessary to control and report transport and associated handling procedures for animals used in μPET studies analyzing the serotonergic system in order to enhance the robustness of conclusions and allow replicability of findings. In view of earlier studies indicating that an increase in hippocampal 5-HT_1A_ receptor expression may be associated with a resilience to stress, it would be of interest to further evaluate 5-HT_1A_ receptor imaging approaches as a candidate biomarker for the vulnerability to stress.

## Introduction

Serotonergic neurotransmission plays a major role in the response to stress exposure, and in the development and manifestation of stress-related mood disorders [1–6]. Among the multitude of serotonergic receptors, 5-HT_1A_ autoreceptors exert a special regulatory function based on a negative feedback system limiting 5-HT release. In addition, 5-HT_1A_ heteroreceptors contribute to postsynaptic serotonergic signaling.

Strong evidence exists that stress exposure can significantly affect serotonergic signaling with a particular impact on 5-HT_1A_ receptor expression in different species [7–11]. An increase in 5-HT_1A_ expression rates has for instance been reported following an unpredictable chronic mild stress paradigm in mice [12].

Here, we addressed the hypothesis that stress-induced alterations in 5-HT_1A_ expression can be determined using molecular imaging with the 5-HT_1A_ receptor radioligand 2′-methoxyphenyl-(*N*-2′-pyridinyl)-*p*-[^18^F]-fluoro-benzamidoethylpiperazine ([^18^F]MPPF). This hypothesis is based on the current state of knowledge about the functional role of 5-HT_1A_ receptors. In the presynaptic localization, 5-HT_1A_ receptors serve an important regulatory function mediating a negative feedback and controlling serotonergic neurotransmission [13, 14]. The hypothalamic-pituitary-adrenal axis interacts closely with serotonergic neurotransmission with corticosteroids exerting direct effects on 5-HT_1A_ function [15, 16]. Thus, it is not surprising that alterations in 5-HT_1A_ and serotonergic signaling have repeatedly been reported associated with stress exposure and have also been linked with resilience to stress [9, 12, 17].

To our knowledge, [^18^F]MPPF positron emission tomography (PET) imaging has not been applied yet in a paradigm with exposure of rodents to stress paradigms. However, our hypothesis is to some extent supported, by alterations in [^18^F]MPPF binding recently reported in a chronic corticosterone depression model, in which the affective state is induced by repeated corticosterone administration [18].

Moreover, alterations in [^18^F]MPPF binding have also been described in other disease models with behavioral alterations, which reflect psychiatric disorders or comorbidities such as increased anxiety or depression [19–21]. Thus, PET-based assessment of 5-HT_1A_ receptors might provide a basis for molecular imaging biomarkers of the regulation of serotonergic neurotransmission in response to stress.

The hypothesis was addressed by [^18^F]MPPF PET imaging in animal groups with mild-to-moderate stress related to either once daily transport to a laboratory with a 1-h stay in the room or once daily transport to the laboratory plus 1 h restraint in a narrow tube. The procedure was repeated for twelve consecutive days. In addition, brain activity patterns were assessed by 2-deoxy-2-[^18^F]fluoro-D-glucose ([^18^F]FDG) μPET. Body weight development, nest building, saccharin preference, serum corticosterone levels, and adrenal gland weight were analyzed in order to obtain additional information about the level of stress and its behavioral and biochemical consequences. Data were compared with those from naive rats.

## Material and methods

### Animals

Thirty-two female Sprague Dawley rats (244.0 ± 10.8 g, Envigo, the Netherlands) were used for this study. All animals were housed individually under controlled environmental conditions (22-24 °C, 45-60% humidity, TECNIPLAST GR 1800 Double Decker) in a 12-h dark-light cycle with ad libitum access to food (Ssniff Spezialdiäten GmbH, Soest, Germany) and tap water. Animals were allowed to acclimatize to the housing facility for 7 days. During this acclimatization phase, animals were habituated to handling by the experimenter with handling sessions every second day for 2-3 min. Once a week, all animals received a clean cage with new bedding material (Lignocel® Select, J. RETTENMAIER & SÖHNE GmbH + Co KG, Rosenberg, Germany) and 14 g of new nesting material (Enviro Dri®, Claus GmbH, Limburgerhof, Germany). During the entire study, the health condition of the animals was checked daily and body weight was measured every third day. The study was approved by the government of Upper Bavaria (license number: AZ 55.2-2532-17-86) and was planned and conducted in line with the German Animal Welfare Act and the EU directive 2010/63/EU. All procedures and reporting were performed according to the ARRIVE guidelines and the Basel Declaration including the 3R concept.

### Stress restraint regimen

**Fig. 1:**
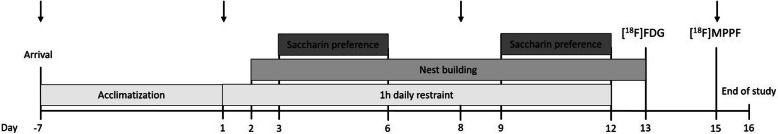
Timeline of study. The black arrow indicates the cage changes with new bedding and nesting material

Rats were randomly allocated to one of three groups (www.randomizer.org): transport + restraint (11), transport (11), and naive (10). For twelve consecutive days, animals in the transport + restraint and the transport groups were removed from their home cage and transported (in a separate transport cage) from the animal facility to a laboratory (distance of < 5 meters). Here, rats of the transport + restraint group were placed in a plexiglass tube with ventilation holes (16 cm in length and 9 cm in diameter) for 1 h (from 10:00 am to 11:00 am) and transport animals were housed individually in a cage with bedding and nesting material. The experimenter was not present in the procedure room during the 1-h exposure phase. Following 1 h, all animals of the transport + restraint and the transport group returned to the animal facility. Naive animals remained in their home cage in the animal facility and were only handled for cage cleaning or weighing during this period. The timeline for the assessment of nest building, saccharin preference test, [^18^F]FDG and [^18^F]MPPF PET imaging, serum corticosterone, and adrenal gland weight of all animals is illustrated in Fig. 1.

### PET acquisition and reconstruction

On day 13, all animals underwent PET imaging with [^18^F]FDG. Small animal PET imaging was performed on a Siemens Inveon DPET scanner (Siemens Medical Solutions, Munich, Germany). Anesthesia was induced by inhalation of isoflurane (4% for induction and 2% for maintenance during preparation, injection, and scanning) supplemented with oxygen at a rate of 1 l/min. [^18^F]FDG was administered via a tail vein cannulation and two rats were simultaneously scanned head-to-head. Rats were fasted overnight to reduce endogenous glucose levels and an emission scan of 15 min was acquired 30 min post tracer injection. The average injected radioactivity was 32.2 ± 7.1 MBq in a volume of 0.5 mL. On day 15, a subset of animals underwent PET imaging with [^18^F]MPPF. Twenty animals were scanned (naive = 7, transport = 6, transport + restraint = 7) with three productions of [^18^F]MPPF. One production of [^18^F]MPPF had an inadequate specific activity and data from these five animals scanned with this batch could not be considered in the analysis resulting in *n* = 5 for all treatment groups. The average injected radioactivity of [^18^F]MPPF was 25.9 ± 6.3 MBq in a volume of 0.5 mL and an emission scan of 50 min was acquired 10 min post tracer injection. The molar activities were in the range of 37-111 GBq/μmol, and the radiochemical purities, as determined by radio-HPLC, were > 96%. All emission scans were either preceded or followed by a 15 min transmission scan using a rotating Cobalt-57 [^57^Co] point source. Data were acquired in list-mode and reconstructed into one frame for [^18^F]FDG and eleven frames (5 × 60 s, 3 × 300 s, 3 × 600 s) for [^18^F]MPPF. All PET data were corrected for attenuation, scatter, and decay. Reconstruction was performed with four ordered-subset-expectation-maximization 3D iterations and 32 maximum-a-posteriori 3D iterations and a zoom factor of one resulting in a final voxel dimension of 0.783 × 0.783 × 0.8 mm.

### PET processing and quantification

Image processing was completed using the PMOD v3.4 software (PMOD Technologies Ltd., Zurich, Switzerland). Tracer-specific PET templates were generated through the averaging of PET images previously aligned by manual co-registration to a digital cryosection-based atlas of the rat brain [22, 23]. Co-registered PET images were then spatially normalized into the space of their respective tracer-specific template by the PMOD brain normalization tool (equal modality; smoothing by 0.8 mm, non-linear warping; 16 iterations, frequency cut-off 3, regularization 1.0, no thresholding) [24]. Volumes of interest (VOIs) were defined as previously described [20]. VOIs for the parietal cortex (16 mm^3^), medial prefrontal cortex (13 mm^3^), thalamus (22 mm^3^), amygdala (23 mm^3^), striatum (15 mm^3^), hypothalamus (11 mm^3^), cerebellum (82 mm^3^), and pons (8 mm^3^) were drawn manually with reference to the cryosection atlas (Supplementary Fig. 1). For visualization purposes, images of mean tracer uptake per treatment group were generated, smoothed with an isotropic Gaussian filter (0.5 mm), and projected onto the cryosection atlas of the rat brain.

[^18^F]FDG uptake was quantified as the standardized uptake value ratio (SUVR) by normalizing tracer uptake in target regions to that of the pons.

For [^18^F]MPPF, the non-displaceable binding potential (BP_nd_) was calculated in target regions from the entire scan [25] using the Logan linear graphical method [26] with the cerebellum as a reference tissue input. Threshold-based automatic VOIs were generated from [^18^F]MPPF uptake for the hippocampus (61 mm^3^) and septum (9 mm^3^) due to the high specific binding in these regions.

### Nest building

Nest building constitutes a non-essential behavior in an animal-facility environment, which can be compromised by distress.

Following the first session in the laboratory, all animals were placed in a clean cage with the addition of new nest material (14 g Enviro Dri®, Claus GmbH, Limburgerhof, Germany). Starting on day 2, the complexity and shape of nests (1 = flat, 2 = slightly curved, 3 = deep) was scored daily between 8 and 9 am for 7 days, at which point all cages were cleaned with the addition of fresh nesting material. Nest scoring was then continued until day 13. The scoring system is illustrated in Supplementary Fig. 2.

### Saccharin preference

The saccharin preference test was used to assess anhedonia-like behavior. A two-bottle testing paradigm was performed over 4 days. On the first and third day, both bottles were filled with tap water. On the second and fourth day, one bottle contained a 0.1% saccharin solution (Aldrich Saccharin ≥ 98%, Sigma-Aldrich Chemie GmbH, Taufkirchen, Germany). To exclude a possible side preference, the placement of the saccharin-containing bottle was alternated from the right (second day) to the left (fourth day) side of the cage. Saccharin preference was analyzed according to a protocol published by Klein et al. [27]. Each bottle contained 300 g of liquid and the consumption over 24 h was measured. The saccharin preference was calculated as saccharin intake/total fluid intake (water + saccharin) × 100. The saccharin test was performed twice to assess possible differences between the early (day 3-6) and late (day 9-12) phase of the restraint stress procedure.

### Termination of study and blood sampling for biochemical parameters

On day 16, all rats were killed by an overdose of pentobarbital (600 mg/kg i.p., Narcoren, Merial GmbH, Hallbergmoos, Germany). Blood samples were obtained by cardiac puncture. Adrenal glands were removed and weighed.

Blood samples were collected in tubes containing Aprotinin (A1153 Sigma-Aldrich, 500 KIU/mL of blood) and left to coagulate for 60 min at room temperature. Afterwards, blood was centrifuged at 2000×*g* for 10 min and the supernatant (serum) was stored at −80 °C. Corticosterone serum levels were quantified using a commercial enzyme-linked immunosorbent assays (ELISA) kit (DEV9922, Demeditec Diagnostic GmbH, Kiel, Germany) according to the manufacturer’s instructions. Optical density was determined using a microplate reader (Gen 5 microplate reader, Biotek; Gen 5 Imager Software, Biotek, Bad Friedrichshall, Germany) set to 450 nm, and a four-parameter logistic (4-PL) curve fit was used to determine sample concentrations in ng/mL.

### Statistics

Statistical analysis was performed with GraphPad Prism (Version 5.04; GraphPad, San Diego, CA, USA) and R version 3.4.3 [28]. Differences between groups were assessed by one-way analysis of variance (ANOVA) followed by a Bonferroni multiple comparison post hoc test or by repeated-measures two-way ANOVA with Tukey HSD post hoc comparisons. Normal distribution was confirmed by a Shapiro-Wilk test. Ordinal data (nest-building scores) were analyzed using the Kruskal-Wallis test followed by Dunn’s multiple comparison post hoc test. Spearman correlation matrix and graphs were calculated and visualized using the R package “corrplot” [29]. Data sets from animals with missing data were not considered for individual correlation analyses. Nest building scores are presented as the median score per day. All other data are shown as mean and standard error of the mean (SEM).

## Results

### Impact on [^18^F]MPPF binding and [^18^F]FDG uptake

**Fig. 2:**
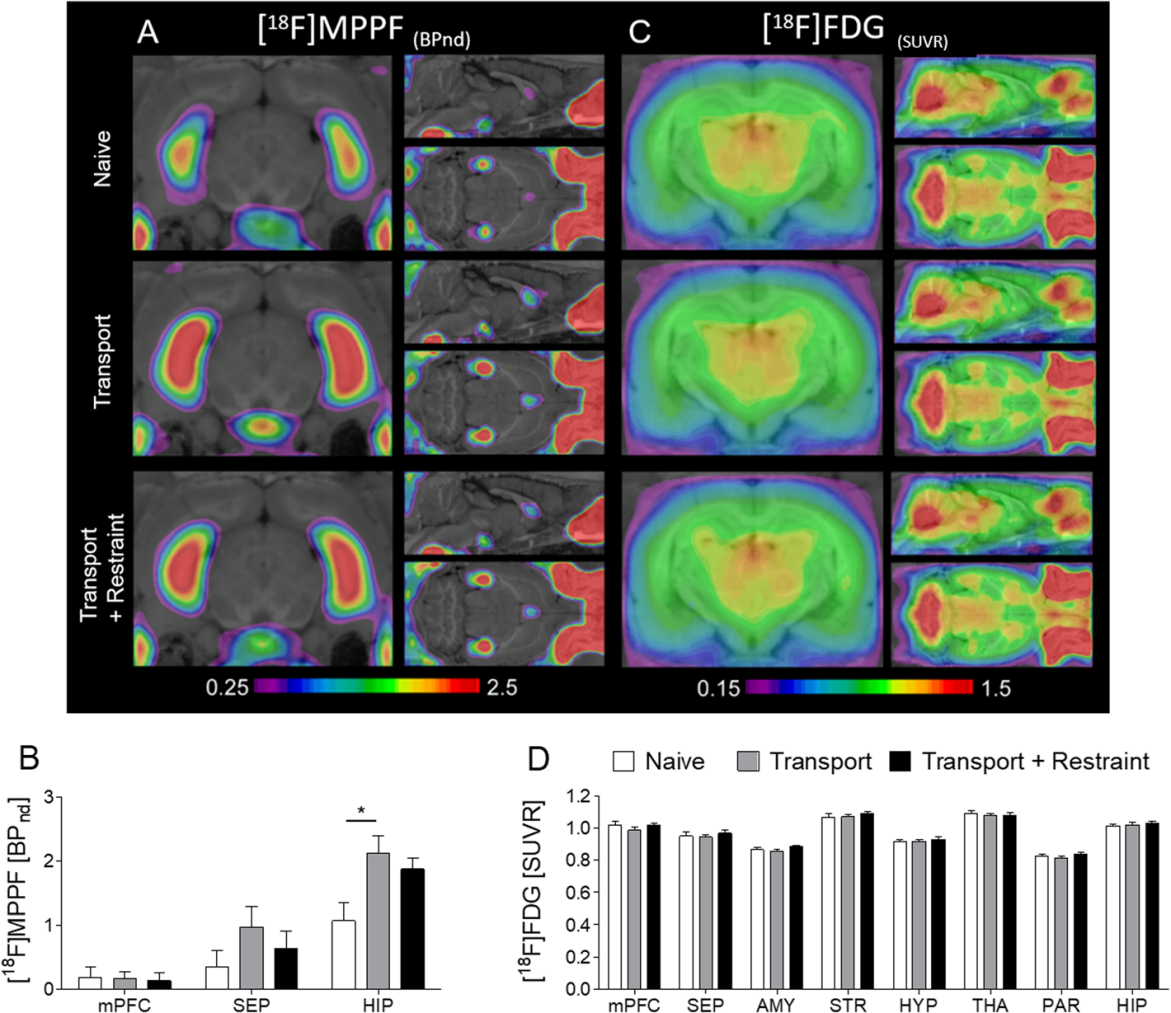
Cerebral glucose metabolism and 5-HT_1A_ receptor availability assessed by [^18^F]FDG and [^18^F]MPPF μPET. **a** Average parametric maps of [^18^F]MPPF binding potential. **b** [^18^F]MPPF binding in the medial prefrontal cortex, septum, and hippocampus. A significant increase of hippocampal [^18^F]MPPF binding became evident in rats with daily transport to the laboratory and a 1-h stay in the laboratory. **c** Averaged [^18^F]FDG metabolic maps. **d** [^18^F]FDG uptake did not significantly differ between groups in any brain region of interest. Data are shown as mean ± SEM. *P < 0.05

5-HT_1A_ receptor availability was assessed by [^18^F]MPPF μPET on day 15, i.e., 3 days following the last exposure to transport or to transport and restraint (Fig. 2a and b). The regions of interest were selected based on information about regional 5-HT_1A_ receptor expression and the average parametric maps of [^18^F]MPPF binding potential (Fig. 2a).

A relevant [^18^F]MPPF binding was detected in all regions of interest comprising the medial prefrontal cortex, septum, and hippocampus (Fig. 2b). In all groups, the highest level of binding was observed in the hippocampus.

In the septum and medial prefrontal cortex, [^18^F]MPPF binding did not differ between groups (*F* (2, 12) = 1.256; *F* (2, 12) = 0.024; Fig. 2b). In rats with transport to the laboratory, hippocampal [^18^F]MPPF binding exceeded that in the transport and restraint group and in the naive group (*F* (2, 12) = 4.759, *p* = 0.030; post hoc *p* = 0.016; Fig. 2b).

Glucose metabolism was studied based on [^18^F]FDG μPET at day 13, i.e., 1 day following the last exposure to transport or to transport and restraint. Averaged [^18^F]FDG metabolic maps for all groups are shown in Fig. 2c.

Comparison between groups did not reveal any relevant group differences in all regions of interest (for all regions: *F*(2, 27) = 0.233-1.790, Fig. 2d).

[^18^F]FDG uptake was quantified with the pons as a reference tissue. We confirmed that [^18^F]FDG uptake in this region did not significantly differ between treatment groups (Supplementary Fig. 3). Two naive rats were excluded from [^18^F]FDG analysis due to a paravenous injection.

### Impact on body weight

**Fig. 3:**
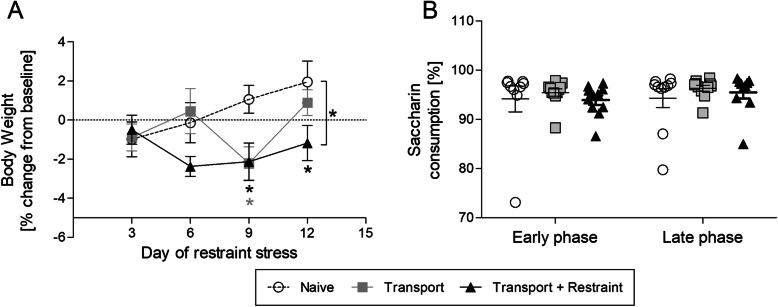
Influence of transport and combined transport and restraint on body weight and saccharin preference. **b** Time course of body weight development illustrated as mean percent change from baseline. **b** Percentage of saccharin consumption in the early phase (day 3-6) and the late phase (day 9-12) of the study. Data are shown as mean ± SEM. *P < 0.05

Body weight was determined on days 1 (= baseline), 3, 6, 9, and 12 of the study immediately prior to transport. The percent weight change at all time points was calculated in relation to baseline level (Fig. 3a).

Variance analysis revealed a significant interaction between the effect of “treatment” and “time” on body weight changes (*F* (6, 87) = 3.045, *p* = 0.0095). A difference to naive control animals became evident at day 9 for the group with transport to the laboratory, and at day 9 and 12 for the group with transport and restraint (post hoc *p* = 0.033; *p* = 0.038; *p* = 0.043).

### Impact on nest building and saccharin preference

The shape and complexity of nests was scored daily from day 2 until day 13. The comparison of scores between groups did not indicate relevant group differences (Supplementary Fig. 4).

The saccharin preference test was performed twice during the study. All groups exhibited a pronounced preference for the saccharin solution at both time points investigated (Fig. 3b). The ratio between water and saccharin consumption was in the same range in all groups, during both the early and the late test phase (early phase: *F* (2, 28) = 0.317; late phase: *F* (2, 28) = 0.568).

### Impact on serum corticosterone and adrenal gland weight

Serum corticosterone levels and adrenal gland weight were determined at the end of the study to assess a possible activation of the hypothalamic-pituitary-adrenal (HPA) gland axis.

Both, corticosterone levels and adrenal gland weight were in the same range in all groups (*F* (2, 29) = 0.6777; *F* (2, 29) = 0.8931; Supplementary Fig. 5A and B).

### Cross correlation between μPET data and other parameters

In order to obtain information about the link between μPET data and clinical, behavioral, or biochemical data, we completed a cross-correlation analysis (Supplementary Fig. 6).

**Fig. 4:**
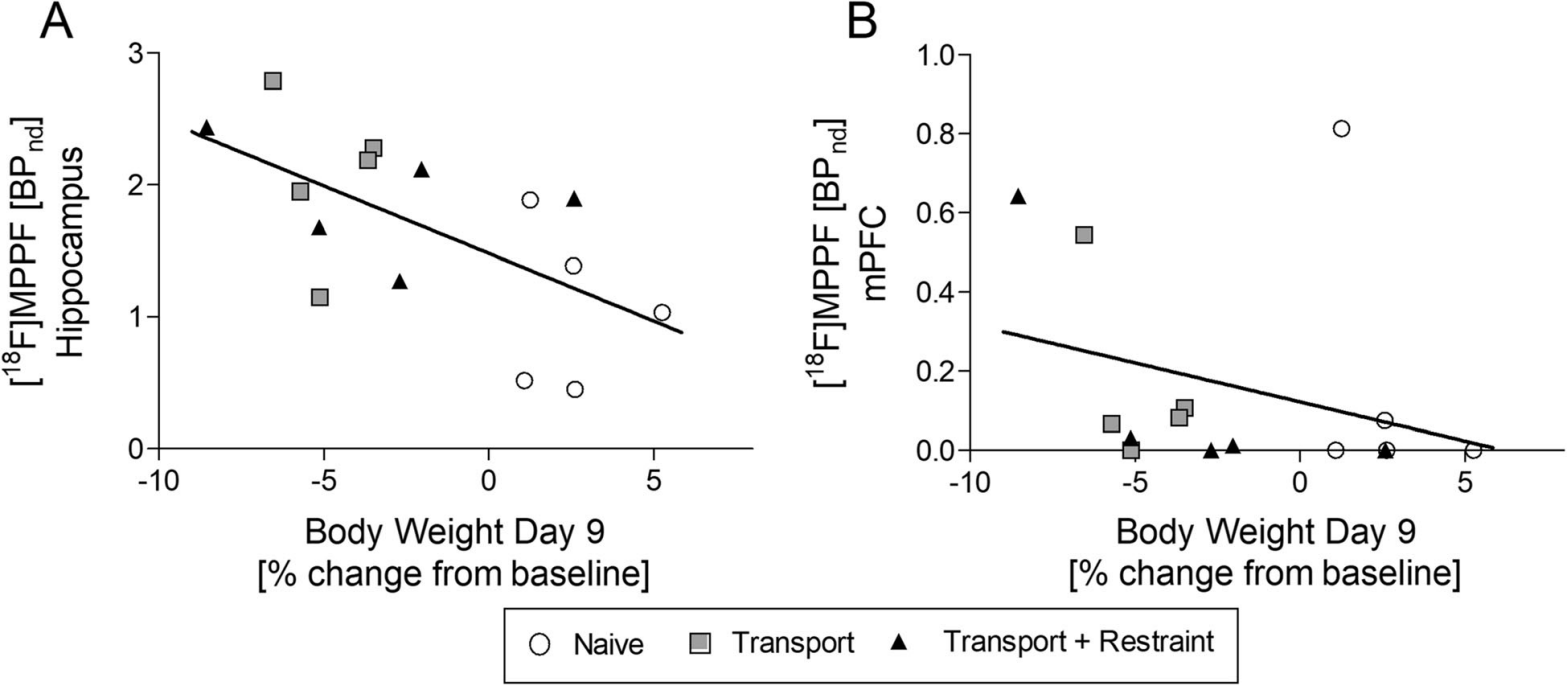
Spearman correlation between body weight at day 9 and [^18^F]MPPF binding in the hippocampus (**a**) and the medial prefrontal cortex (**b**)

A moderate correlation was shown between body weight change at day 9 and [^18^F]MPPF binding in the hippocampus and medial prefrontal cortex (*r* = −0.64, *p* = 0.01; *r* = −0.50, *p* = 0.057; Fig. 4).

## Discussion

μPET imaging revealed alterations in hippocampal 5-HT_1A_ receptor-mediated serotonergic signaling as a consequence of a twelve-day study with daily transport and 1-h stay in a laboratory. Unexpectedly, transport in combination with 1-h restraint as a traditional stress paradigm did not result in additive or synergistic effects. This finding might be due to a possible ceiling effect with a level of stress caused by the transport procedure that surpasses the potential effects of restraint. Brain activity patterns assessed by [^18^F]FDG were neither affected by daily transport nor by combined transport and restraint.

It is highly interesting to note that repeated transport of animals to a laboratory with limited handling, i.e., placing the animals in a transport cage and then in a new cage for an hour, is sufficient to induce significant changes in hippocampal [^18^F]MPPF binding. Regarding the reasons for the alterations of [^18^F]MPPF binding in these animals, there are different possible explanations. Alterations in [^18^F]MPPF binding may be related to the novelty of the experience on experimental days rather than a negative experience in the sense of distress. However, in this context, it needs to be considered that body weight also declined in this group reaching a significant difference to naive animal at day nine of the exposure phase, and that body weight data correlated with alterations in [^18^F]MPPF binding. While data from the end of the exposure phase seem to indicate an additive or synergistic effect, the drop in body weight and trough level observed at day 9 of the procedure was almost identical in both groups again suggesting that the transport procedure alone already exerts a ceiling effect. The fact that maximum effects were observed at day nine of the exposure phase indicates that as expected there is a delay in measurable alterations in body weight and that a habituation is observed toward the end of the twelve-day exposure phase.

Considering that body weight loss can reflect a compromised well-being and increased distress levels in laboratory rodents [30, 31], these findings support a mild to moderate level of distress in both groups and thus a link between increased hippocampal [^18^F]MPPF binding and distress. A respective link has previously been suggested based on our findings in epilepsy models, in which increased [^18^F]MPPF binding correlated with behavioral comorbidities, but did not correlate with seizure frequency or parameters [19, 20]. Theoretically, stress contagion, which has been reported for rats [32], might have contributed as the animal groups with transport or combined transport and restraint stayed in the same laboratory room. The term stress contagion has previously been introduced for the observation that animals exposed to stress may under specific circumstances increase the stress level of other animals in the same room [32]. Concerning our findings, it is, however, rather unlikely that stress contagion had a relevant impact on the data from the group with transport considering that no significant increase in [^18^F]MPPF binding became evident in rats with repeated restraint.

Taken together, our data indicate that a 12-day experimental phase with daily transport to a laboratory and return to the animal facility after 1 h causes a level of distress resulting in a transient decline in body weight and an increase in 5-HT_1A_ receptor binding. In this context, it needs to be considered that based on the timing of [^18^F]MPPF imaging with analysis, 3 days following the transport and restraint exposure phase respective data indicate longer-lasting effects of stress, whereas the body weight analysis during the exposure phase confirms immediate effects.

The [^18^F]MPPF data need to be considered in the context of our findings for biochemical parameters concerning HPA axis activation. Serum corticosterone increases have been reported in laboratory rodents following different handling procedures with levels varying depending on techniques and strains [33, 34]. Higher levels of corticosterone have for instance been reported in Sprague Dawley than in Lewis rats following a 7-day handling procedure, but not following a 14-day handling procedure indicating that Sprague Dawley rats may not only respond more intensely but also habituate over time [34]. In the present study, we took care to habituate the rats to handling procedures before the study in order to be able to assess the impact of transport and exposure to a laboratory environment. To our knowledge, a respective impact has not been studied previously. Neither serum corticosterone nor adrenal gland weight was affected in the groups with transport or transport combined with restraint. In this context, it needs to be taken into account that we, considering the study aim, avoided invasive blood sampling during the study phase, and therefore serum corticosterone levels are only available from the final day of the study, i.e., the 4th day following the last “transport to the laboratory” or “restraint session.” Thus, we can exclude a persistent increase in HPA axis activation, but the data of course do not argue against acute and transient distress associated with the repeated exposure to transport and the laboratory environment.

The adrenal gland weight of rats can increase following prolonged exposure to intense stressors [35, 36], thereby confirming a pronounced and lasting activation of the HPA axis. As rats in the present study were only exposed to mild-to-moderate stress situations, we did not expect an increase in adrenal gland weight. Thus, taken together the body weight data in combination with the biochemical data indicate that the stress associated with daily transport and exposure to the laboratory is rather mild-to-moderate not resulting in a persistent HPA axis activation. In view of this conclusion, the increase in 5-HT_1A_ receptor binding is all the more remarkable. The result indicates that transport and environmental conditions as well as handling procedures need to be carefully controlled and reported for animals used in μPET studies analyzing the serotonergic system in order to guarantee the robustness of the conclusions.

As already stated in the introduction, exposure to stressful situations can alter serotonergic signaling with a particular effect on 5-HT_1A_ receptor expression rates [7–12]. In mice exposed to unpredictable chronic mild stress elevated 5-HT_1A_ receptor mRNA and protein levels have been described in the prefrontal cortex and hippocampus [12]. An induction of 5-HT_1A_ receptor expression was also evident in rat subgroups with exposure to a chronic mild stress paradigm [37]. The enhanced binding of the 5-HT_1A_ receptor ligand [^18^F]MPPF that we observed in the hippocampus of rats with repeated exposure to transport and the laboratory environment is thus in line with an increased hippocampal 5-HT_1A_ expression rate reported in response to mild stress paradigms. Interestingly, Zurawek and colleagues found that a respective upregulation of 5-HT_1A_ receptors only occurred in rats with a resilient phenotype, and not in rats with high vulnerability to stress [37]. Thus, the regulation of 5-HT_1A_ receptors in the hippocampus might serve as an important molecular stress coping mechanism, which allows maintaining or restoring the homeostatic balance in serotonergic neurotransmission. However, data interpretation also needs to take into account that [^18^F]MPPF binding can also increase as a consequence of a reduction in endogenous serotonin concentrations and an altered occupancy and competition at 5-HT_1A_ receptors [38].

In addition, future studies would be of interest, which compares tracer binding to 5-HT_1A_ receptors in subgroups of rats, which exhibit a different vulnerability to stress with differences in the development of behavioral comorbidities. Respective studies may further confirm 5-HT_1A_ receptor PET data as a biomarker candidate to assess the vulnerability state following exposure to stressful or traumatic situations and the risk for long-term consequences such as stress-associated mood disorders. In this context, it will be important to also compare the consequences of stressors with a different intensity level.

In view of the data from the “transport group,” it was a surprising outcome that the combination of transport and restraint failed to exert an additive or synergistic effect on binding to hippocampal 5-HT_1A_ receptors. The sub-chronic restraint procedure is a traditional stress paradigm, which has been frequently applied in stress research [39, 40]. Depending on the procedure, immobilization, and restraint can even cause the development of stress-induced gastric lesions and ulcers [41]. The paradigm that we have selected for the present study has previously been classified as an intermediate-intensity stressor in a study, which confirmed that a 14-day restraint procedure exerted milder effects on the HPA axis function than an immobilization paradigm with taping of the limbs to metal mounts [42]. Thus, the lack of a significant effect on 5-HT_1A_ receptors may be related to a relatively low level of stress associated with the restraint procedure. In comparison, rats may even feel more protected in a restraint tube than in a cage, when exposed to a new environment. However, in this context, one needs to consider that we did not confirm a direct significant difference between the rat group with transport or combined transport and restraint.

Stress exposure may limit the response to positive and rewarding stimuli such as the offer of a sweet solution, which laboratory rodents generally prefer over water [43]. Transient drops in saccharin preference have been described as a consequence of physical, emotional, and restraint stress situations in rats [43, 44]. In the present study, the lack of relevant alterations in rats with repeated transport or combined transport and restraint argues against the induction of anhedonia-associated behavior in both groups, again confirming that the stress associated with the procedures has been rather mild to moderate.

Previous studies reported early changes in [^18^F]FDG uptake in response to acute immobilization stress [45], uncontrollable and unpredictable foot-shock stress [46], and a prolonged and more intense restraint stress protocol [47]. Based on earlier findings, these stress paradigms have been classified as rather severe reflected by pronounced alterations in different parameters that have been studied in the context of stress assessment [48–50]. Thus, the lack of alterations in metabolic activity 1 day following the end of the 12-day restraint stress phase might further confirm the classification as a rather mild-to-moderate procedure.

Taken together the most important finding is that exposure to a common procedure of transporting animals from one room to another with a 1-h stay in a laboratory room is sufficient to cause alterations in the serotonergic system, which can be visualized by [^18^F]MPPF PET imaging, and a transient decline in body weight. On one hand, these findings point to the fact that respective factors need to be carefully controlled and reported in order to guarantee the robustness and replicability of respective imaging studies. On the other hand, the data suggest imaging of 5-HT_1A_ receptor binding as a biomarker candidate for assessment of distress. In this context, the limitations of a single study with specific conditions in the laboratory and the animal facilities need to be considered. As also demonstrated by our findings, the environmental, and procedural conditions can have a major impact on the outcome. Thus, further studies will be necessary to assess the validity of 5-HT_1A_ receptor binding as a potential biomarker of distress under different laboratory and animal facility conditions. In this context, it will also be of particular relevance to analyze the impact of the rat strain, age, and sex, and to test whether the findings translate to other species.

Moreover, as a ceiling effect caused by the transport procedure might have masked effects of the restraint procedure, it is of additional interest to test the effect of a restraint procedure in the home cage of the animals.

Finally, as mentioned above, receptor binding in PET imaging studies can be influenced by, both, changes in receptor expression and in the concentration of the endogenous ligand. Therefore, additional studies with immunohistochemistry and microdialysis assessing receptor expression and serotonin concentrations are necessary for respective conclusions.

## Conclusions

Our findings demonstrate that mild-to-moderate stress associated with daily transport and exposure to a laboratory environment can trigger significant alterations in hippocampal binding of the 5-HT_1A_ receptor ligand [^18^F]MPPF. This finding indicates that utmost care is necessary to control and report transport and environmental conditions, and all handling procedures for animals used in μPET studies analyzing the serotonergic system in order to enhance the robustness of the conclusions and allow replicability of the findings.

In view of earlier studies suggesting that an increase in hippocampal 5-HT_1A_ receptor expression may be associated with a resilience to stress, it would be of interest to further evaluate 5-HT_1A_ receptor imaging approaches as a candidate biomarker for the vulnerability to stress and the risk for the development of stress-associated mood disorders.

## Supplementary information

**Additional file 1: Supplementary Fig. 1.** Analyzed brain regions. Definition of target regions comprising medial prefrontal cortex (dark blue, A), septum (dark green, B and G), striatum (orange, B), hippocampus (light blue, C and F), parietal cortex (purple, E), amygdala (red, E and F), thalamus (pink, E, F and G ), hypothalamus (light green, E, F and G) and the pons as reference (light yellow, D and G) in the cryosection atlas of the rat brain in coronal and sagittal slices. **Supplementary Fig. 2.** Illustration of nest complexity scoring system. (A) Score 0, (B) Score 1 (flat), (C) Score 2 (slightly curved), (D) Score 3 (deep). **Supplementary Fig. 3.** [^18^F]FDG uptake in Pons. [^18^F]FDG uptake was quantified as the standardized uptake value ratio (SUVR) by normalizing tracer uptake in target regions to that of the pons. [^18^F]FDG uptake in this region did not significantly differ between treatment groups (F(2,27) = 0.07956, p = 0.9237). **Supplementary Fig. 4.** The influence of daily restraint stress on nest complexity scores. Time course of the median nest scores for each treatment group. The black arrow indicates the introduction of new nesting material. Nest scores did not differ in a significant manner between groups. **Supplementary Fig. 5.** Effect of chronic restraint stress on serum corticosterone levels and adrenal gland weight. (A) Serum corticosterone levels and (B) adrenal gland weight were assessed at the end of the study. Graphs show individual values and mean ± SEM. Data were in the same range in all groups. **Supplementary Fig. 6.** Correlation matrix illustrating cross-correlation between PET, behavioral, physiological and biochemical parameters.

## Data Availability

The datasets generated or analyzed during the current study will be available in the severity assessment repository of the DFG research unit 2591 [https://severity-assessment.de].

## Abbreviations

ANOVA: Analysis of variance
BP_nd_: Non-displaceable binding potential
ELISA: Enzyme-linked immunosorbent assays
[^18^F]FDG: 2-deoxy-2-[^18^F]fluoro-D-glucose
[^18^F]MPPF: 2′-methoxyphenyl-(N-2′-pyridinyl)-p-[^18^F]fluoro-benzamidoethylpiperazine
HPA: Hypothalamic-pituitary-adrenal
PET: Positron emission tomography
SEM: Standard error of the mean
SUV: Standardized uptake value
SUVR: Standardized uptake value ratio
VOI: Volume-of-interest
