# Supplementary material for: [18F]MPPF and [18F]FDG μPET imaging in rats: impact of transport and restraint stress
Source: EJNMMI Res. 2020 Sep 29;10:112. doi: 10.1186/s13550-020-00693-3 (PMC7524912; doi:10.1186/s13550-020-00693-3)
Supplement: Supplementary file 1 — Additional file 1: Supplementary Fig. 1. Analyzed brain regions. Definition of target regions comprising medial prefrontal cortex (dark blue, A), septum (dark green, B and G), striatum (orange, B), hippocampus (light blue, C and F), parietal cortex (purple, E), amygdala (red, E and F), thalamus (pink, E, F and G ), hypothalamus (light green, E, F and G) and the pons as reference (light yellow, D and G) in the cryosection atlas of the rat brain in coronal and sagittal slices. Supplementary Fig. 2. Illustration of nest complexity scoring system. (A) Score 0, (B) Score 1 (flat), (C) Score 2 (slightly curved), (D) Score 3 (deep). Supplementary Fig. 3. [18F]FDG uptake in Pons. [18F]FDG uptake was quantified as the standardized uptake value ratio (SUVR) by normalizing tracer uptake in target regions to that of the pons. [18F]FDG uptake in this region did not significantly differ between treatment groups (F(2,27) = 0.07956, p = 0.9237). Supplementary Fig. 4. The influence of daily restraint stress on nest complexity scores. Time course of the median nest scores for each treatment group. The black arrow indicates the introduction of new nesting material. Nest scores did not differ in a significant manner between groups. Supplementary Fig. 5. Effect of chronic restraint stress on serum corticosterone levels and adrenal gland weight. (A) Serum corticosterone levels and (B) adrenal gland weight were assessed at the end of the study. Graphs show individual values and mean ± SEM. Data were in the same range in all groups. Supplementary Fig. 6. Correlation matrix illustrating cross-correlation between PET, behavioral, physiological and biochemical parameters. [file 13550_2020_693_MOESM1_ESM.docx]

**Supplementary material**

**[^18^F]MPPF and [^18^F]FDG µPET imaging in rats: impact of transport and restraint stress**

**Verena Buchecker^1*^, Ann-Marie Waldron^1*^, R. Maarten van Dijk^1^, Ines Koska^1^, Matthias Brendel^2^, Barbara von Ungern-Sternberg^2^, Simon Lindner^2^, Franz Josef Gildehaus^2^, Sibylle Ziegler^2^, Peter Bartenstein^2^, Heidrun Potschka^1#^**

**1.** Institute of Pharmacology, Toxicology, and Pharmacy, Ludwig-Maximilians-University, Munich, Germany.

**2.** Department of Nuclear Medicine, University Hospital of Munich, Ludwig-Maximilians-University, Munich, Germany.

*
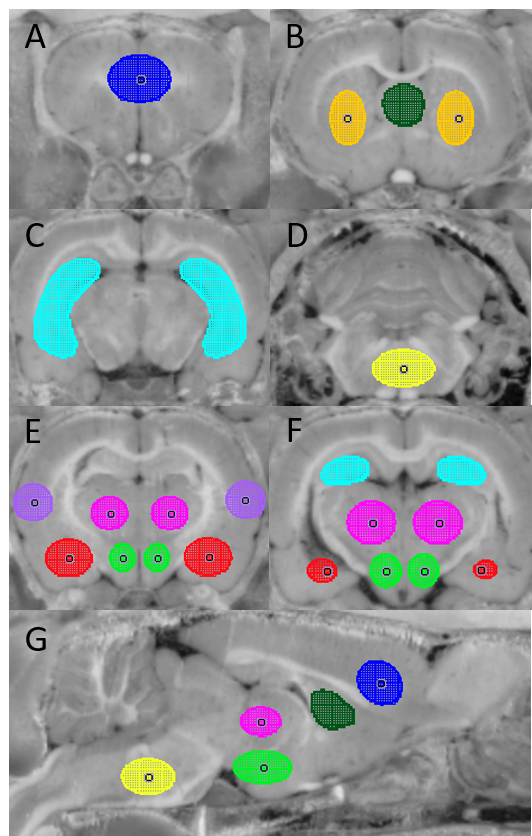
*

*Supplementary Fig. 1. Analyzed brain regions. Definition of target regions comprising medial prefrontal cortex (dark blue, A), septum (dark green, B and G), striatum (orange, B), hippocampus (light blue, C and F), parietal cortex (purple, E), amygdala (red, E and F), thalamus (pink, E, F and G ), hypothalamus (light green, E, F and G) and the pons as reference (light yellow, D and G) in the cryosection atlas of the rat brain in coronal and sagittal slices.*

*
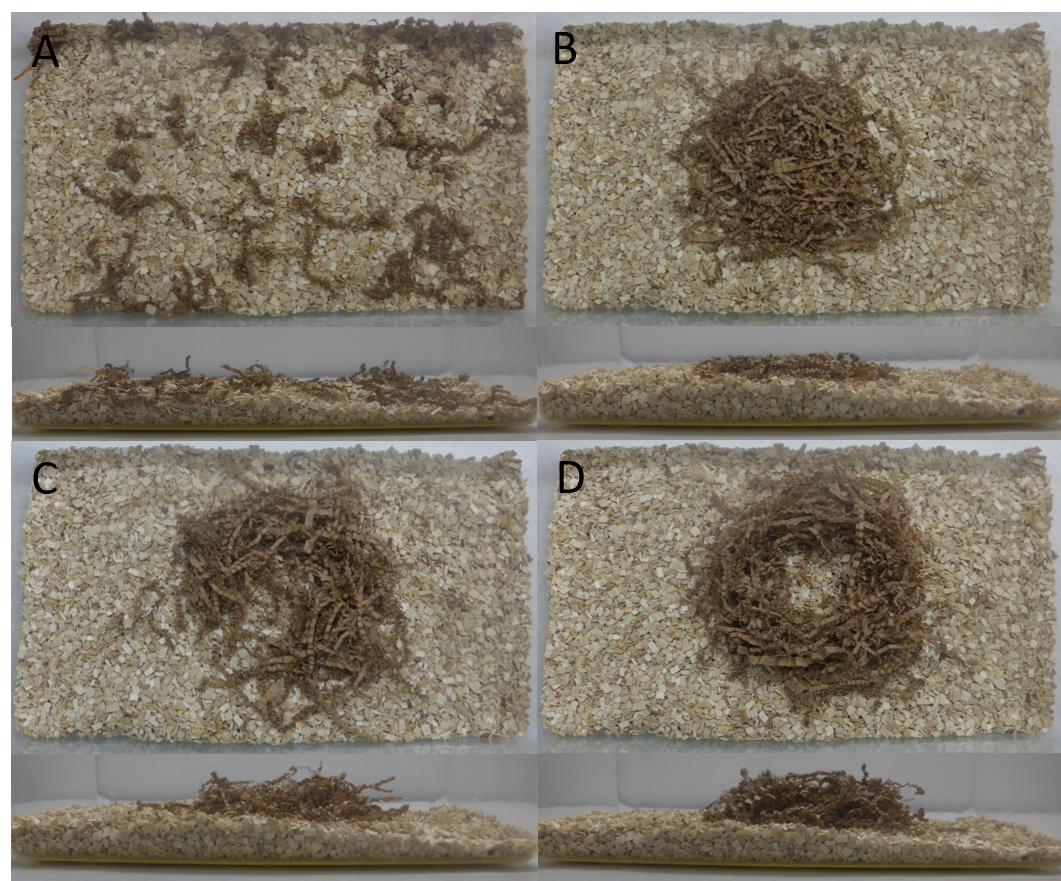
*

*Supplementary Fig. 2. Illustration of nest complexity scoring system. (A) Score 0, (B) Score 1 (flat), (C) Score 2 (slightly curved), (D) Score 3 (deep).*

*
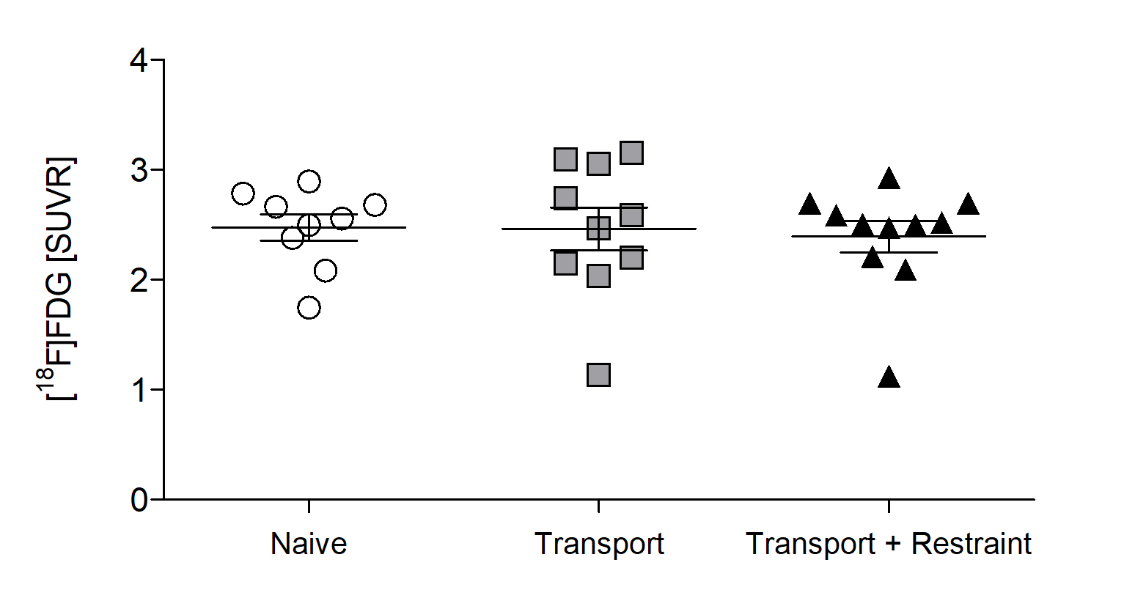
*

*Supplementary Fig. 3. [^18^F]FDG uptake in Pons. [^18^F]FDG uptake was quantified as the standardized uptake value ratio (SUVR) by normalizing tracer uptake in target regions to that of the pons. [^18^F]FDG uptake in this region did not significantly differ between treatment groups (F(2,27) = 0.07956, p = 0.9237)*

*
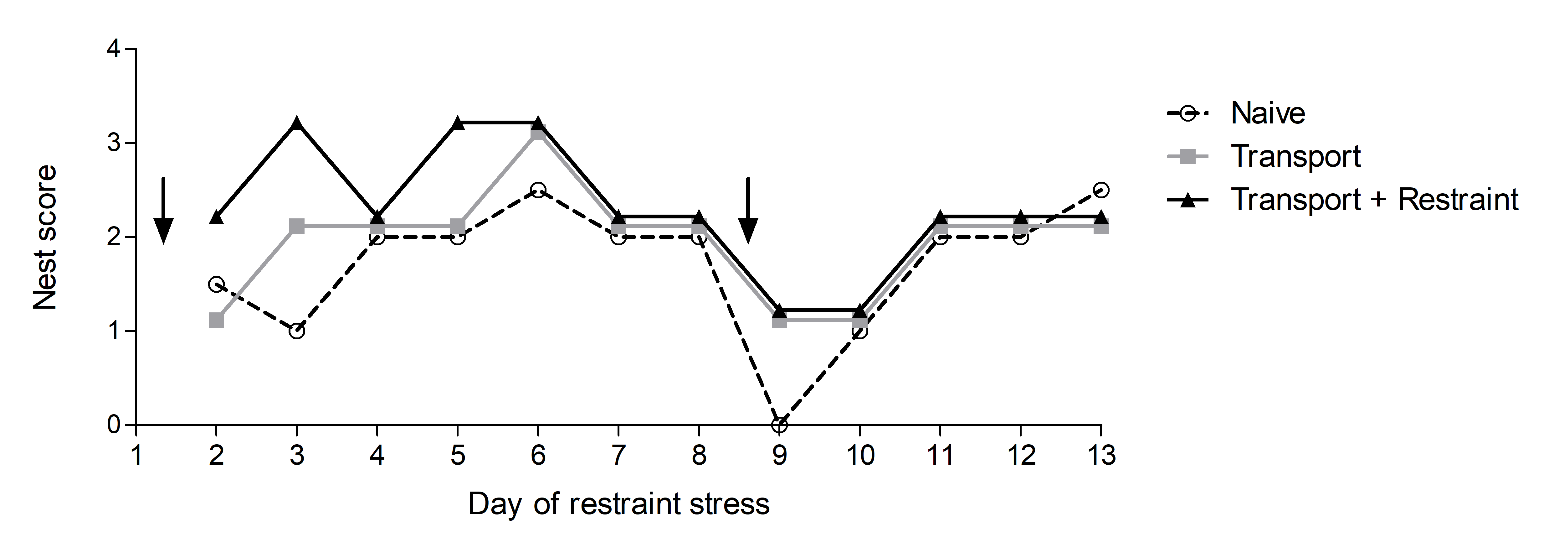
*

*Supplementary Fig. 4. The influence of daily restraint stress on nest complexity scores. Time course of the median nest scores for each treatment group. The black arrow indicates the introduction of new nesting material. Nest scores did not differ in a significant manner between groups.*

*
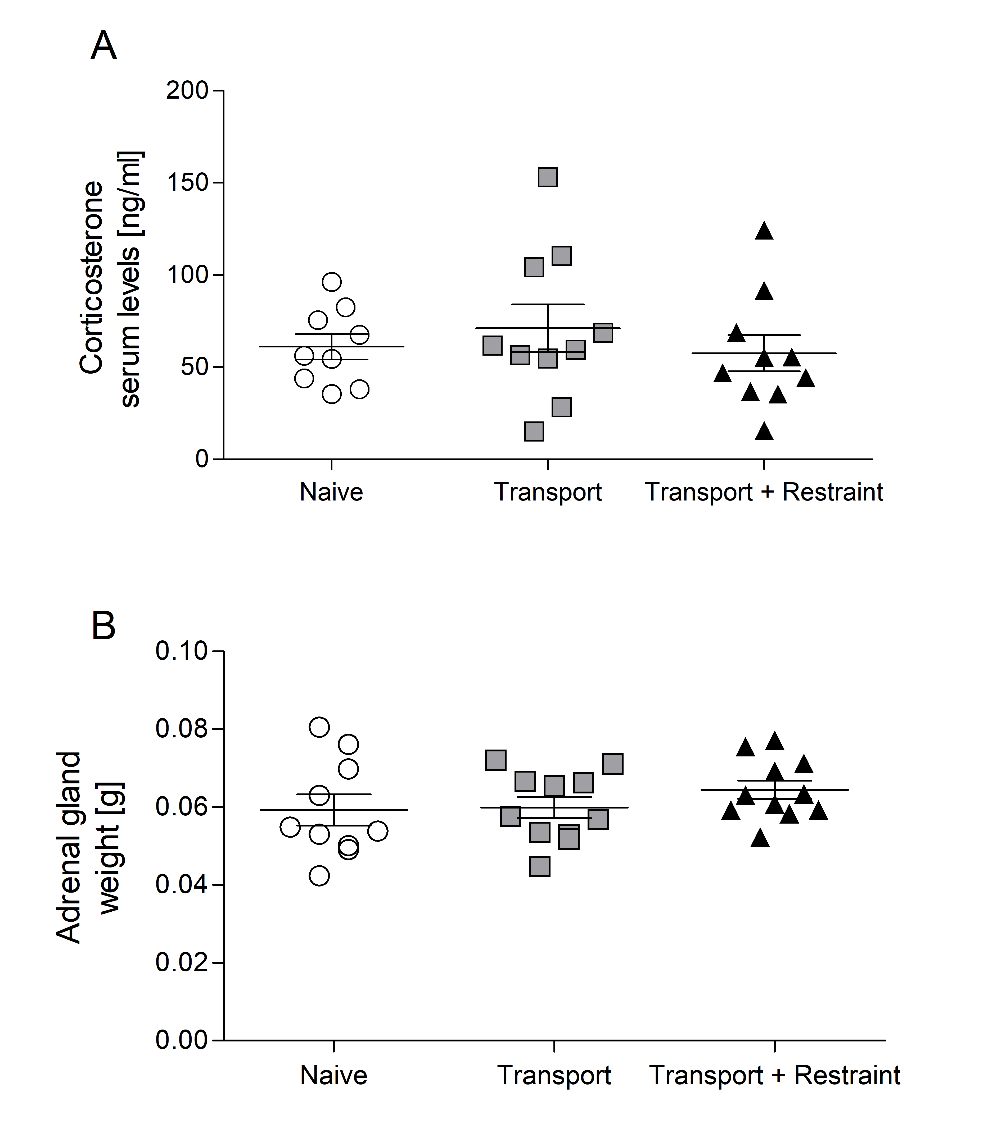
*

*Supplementary Fig. 5. Effect of chronic restraint stress on serum corticosterone levels and adrenal gland weight. (A)* *Serum corticosterone levels and (B) adrenal gland weight were assessed at the end of the study. Graphs show individual values and mean ± SEM. Data were in the same range in all groups.*


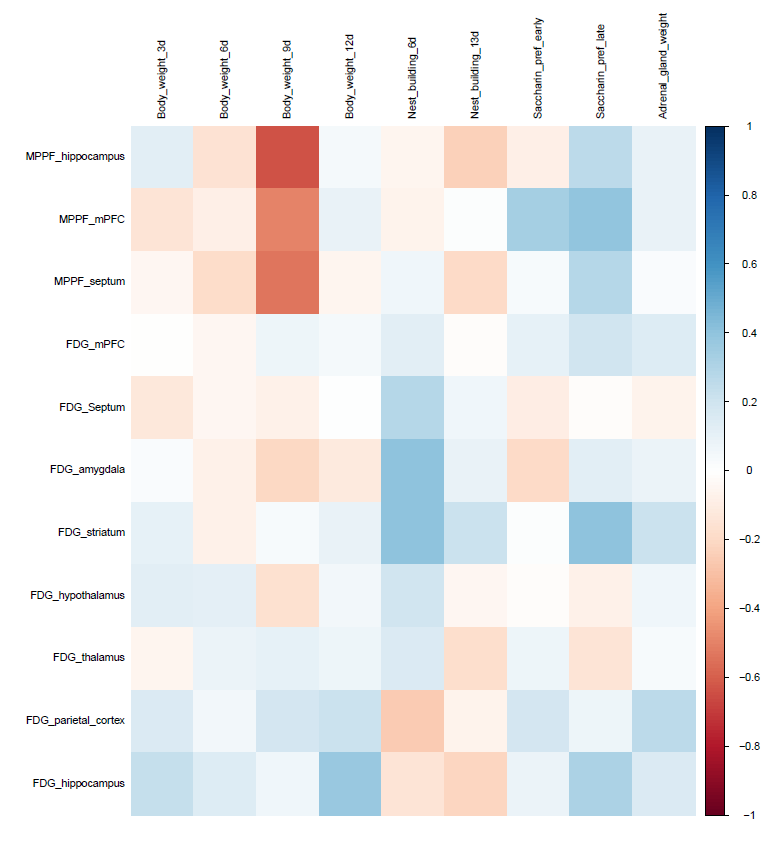


*Supplementary Fig. 6. Correlation matrix illustrating cross-correlation between PET, behavioral, physiological and biochemical parameters.*
